# Dapagliflozin associates with heart rate variability decline in T2DM patients on GLP-1 receptor agonist therapy: a prospective observational study

**DOI:** 10.3389/fendo.2026.1809146

**Published:** 2026-05-11

**Authors:** Ying Wang, Hongning Li, Zhongyuan Zhang, Xiaocen Kong, Huiqin Li, Weichun Qian, Jianhua Ma

**Affiliations:** 1 Department of Endocrinology, Affiliated Nanjing Hospital of Nanjing Medical University, Nanjing, China; 2 Department of Endocrinology, Lishui District People’s Hospital, Nanjing, China; 3 Department of Endocrinology, Nanjing Tongren Hospital, Nanjing, China; 4 Department of Cardiology, Nanjing First Hospital, Nanjing Medical University, Nanjing, China

**Keywords:** cardiac autonomic neuropathy, dapagliflozin, GLP-1 RAs, heart rate variability, T2DM

## Abstract

**Background:**

Glucagon-like peptide-1 receptor agonists (GLP-1 RAs) are associated with increased heart rate (HR) and reduced heart rate variability (HRV) in patients with type 2 diabetes mellitus (T2DM). While sodium-glucose co-transporter 2 inhibitors (SGLT2i) may exert beneficial effects on cardiac autonomic function, it remains uncertain whether baseline SGLT2i use is associated with attenuation of GLP-1 RA-related HRV decline in T2DM.

**Methods:**

In this prospective observational study, 45 patients with T2DM were divided into two groups according to pre-study dapagliflozin use: a dapagliflozin-naïve group (Control group, n=22) and a dapagliflozin-exposed group (DAPA group, n=23). All participants subsequently received GLP-1 RA therapy for 12 weeks. Changes in HRV parameters were assessed by 24-hour ambulatory electrocardiography before and after treatment. Between-group differences were further evaluated using analysis of covariance (ANCOVA), inverse probability of treatment weighting (IPTW), and multivariable linear regression models.

**Results:**

After 12 weeks of GLP-1 RA therapy, the Control group showed significant reductions in SDNN, SDANN, RMSSD, pNN50, and lnHF, together with increases in lnLF and the lnLF/lnHF ratio, whereas no significant within-group changes in HRV indices were observed in the DAPA group. In unadjusted between-group analyses, several HRV parameters differed significantly between groups. After covariate adjustment, significant between-group differences remained for SDNN, SDANN, lnHF, and the lnLF/lnHF ratio. IPTW-weighted sensitivity analyses yielded consistent findings. In multivariable regression, baseline dapagliflozin use was most clearly associated with a more favorable change in SDNN.

**Conclusion:**

In patients with T2DM, dapagliflozin use was associated with a decline in HRV during GLP-1 RA therapy; these findings are hypothesis-generating and require confirmation in larger prospective studies.

## Introduction

T2DM is a complex metabolic disorder characterized by hyperglycemia and associated with a high risk of cardiovascular complications; CAN is a severely debilitating yet under diagnosed condition in patients with diabetes (1). Multi-factorial risk factors, including obesity, hypertension, and hyper-lipidemia, are associated with the development of CAN in T2DM (2). Indices of HRV, a non-invasive quantitative marker of cardiac autonomic function, are composed of different frequency and time domain components attributed to parasympathetic and sympathetic activity, which can predict the risk of cardiovascular morbidity and mortality (3).

In the current therapeutic landscape, GLP-1 RAs have become a first-line choice for the treatment of T2DM with Atherosclerotic Cardiovascular Disease (ASCVD) due to their excellent glucose-lowering effects and clear benefits in cardiovascular health. Despite their benefits, clinical observations and studies indicate that GLP-1 RAs are consistently associated with a mild yet sustained increase in average heart rate and a reduction in HRV (4, 5). This phenomenon points to complex effects on cardiac autonomic balance, the clinical relevance of which remains incompletely understood. Therefore, an important consideration in their use is how to minimize potential negative autonomic consequences while maintaining therapeutic efficacy.

By contrast, beyond their glucose-lowering effects, SGLT2i exhibit pronounced cardio-protective and reno-protective properties. Evidence suggests these benefits may be partially mediated by favorable modulation of cardiac autonomic regulation. Proposed mechanisms include the modulation of myocardial energy metabolism, attenuation of inflammation and fibrosis, and enhancement of HRV, thereby promoting the restoration of sympathetic-vagal balance (6). Clinically, these agents ameliorate dysfunction linked to cardiac autonomic neuropathy and reduce vasovagal syncope episodes in patients with diabetes (7).

However, with the growing clinical adoption of combined SGLT2i and GLP-1 RAs therapy, a critical knowledge gap emerges. Current evidence does not conclusively determine whether SGLT2i can counteract potential GLP-1 RA-induced alterations in cardiac autonomic function. Specifically, it remains unknown if pretreatment with an SGLT2i (e.g., dapagliflozin) confers a protective effect that mitigates the decline in HRV associated with the initiation of GLP-1 RAs therapy.

Against this background, this study aims to elucidate the potential modulating effect of dapagliflozin pretreatment on GLP-1 RAs-induced changes in HRV in patients with T2DM. We hypothesize that such pretreatment will confer a protective effect, resulting in a more favorable HRV profile (i.e., lesser decline or better preservation of HRV parameters) following GLP-1 RAs initiation, compared to controls without SGLT2i pretreatment. To test this hypothesis, we designed a prospective observational study employing systematic 24-hour ambulatory electrocardiography for HRV assessment. A comparison of time- and frequency-domain parameters between the DAPA group and the control group will be conducted at baseline and after a 3-month course of GLP-1 RAs therapy. This investigation is expected to yield novel data for optimizing therapeutic strategies in T2DM.

## Methods

### Study design

This study was a prospective observational cohort study. It was designed to assess the association between dapagliflozin use and changes in heart rate variability (HRV) during GLP-1 receptor agonist (GLP-1 RA) therapy in patients with type 2 diabetes mellitus (T2DM).

### Study population

A total of 45 patients with T2DM who attended the Department of Endocrinology, Nanjing First Hospital, Nanjing Medical University, between January 2023 and January 2025 were enrolled.

### Exposure definition and group allocation

Patients were divided into two groups according to whether they had regularly used dapagliflozin (10 mg/day for at least 12 weeks) before initiation of GLP-1 RA therapy. The DAPA group included patients who had received dapagliflozin before GLP-1 RA therapy, whereas the Control group included patients who had not used any SGLT2 inhibitor before GLP-1 RA therapy.

The decision to use dapagliflozin before study enrollment was made by the physician based on clinical assessment, in accordance with local guidelines and routine standards of care for T2DM. Common considerations included the need for additional glycemic control and the presence of atherosclerotic cardiovascular disease, heart failure, chronic kidney disease, or obesity. There was no pre-specified protocolized decision pathway for dapagliflozin initiation, reflecting real-world clinical practice.

### GLP-1 receptor agonist treatment

After enrollment, all participants initiated GLP-1 RA therapy based on their existing antidiabetic regimen and continued treatment for 12 weeks. The GLP-1 RAs used in this study included polyethylene glycol loxenatide and semaglutide. The therapeutic dose of polyethylene glycol loxenatide was 0.2 mg every week, whereas semaglutide was administered at 0.5 mg every week.

### Eligibility criteria

Inclusion Criteria (1): Age ≥ 18 years (2). Meet the diagnostic criteria for T2DM according to the World Health Organization (WHO) or the American Diabetes Association (ADA) (3). Patients were required to initiate GLP-1 RAs treatment to optimize blood glucose control based on clinical assessment (4). Sinus rhythm, able to cooperate with 24-hour dynamic electrocardiogram monitoring (5). Voluntarily participate and sign informed consent.

Exclusion Criteria (1): Type 1 diabetes, gestational diabetes, or other special types of diabetes (2). Presence of severe arrhythmias (such as atrial fibrillation, frequent ventricular premature beats >10%), second-degree or higher atrioventricular block, or sick sinus syndrome (3). History of unstable angina, acute myocardial infarction, stroke, or heart failure hospitalization within the past 6 months (4). Known severe structural heart disease (such as severe valvular disease, hypertrophic cardiomyopathy) (5). Currently using medications that may significantly affect heart rate and HRV (such as non-dihydropyridine calcium channel blockers, antiarrhythmic drugs, tricyclic antidepressants, cholinesterase inhibitors, etc.). Users of β-blockers with stable doses for ≥1 month may be included (6). Presence of severe liver or kidney dysfunction (such as alanine aminotransferase or aspartate aminotransferase >3 times the upper limit of normal, estimated glomerular filtration rate <45 mL/min/1.73 m²) (7). Presence of uncontrolled hyperthyroidism or hypothyroidism (8). Presence of systemic diseases that may affect autonomic nerve function, such as tumors, severe infections, autoimmune diseases, etc. (9) Pregnant or breastfeeding women.

### Laboratory assessments

Venous blood samples were collected in the morning after an overnight fast at baseline (before initiation of GLP-1 RA therapy) and again after 12 weeks of treatment.

Glucose metabolism indicators included glycated hemoglobin A1c (HbA1c), fasting and 2-hour postprandial blood glucose (after a standard steamed-bread meal), insulin, C-peptide, and glucagon. Lipid metabolism indicators included total cholesterol, triglycerides, high-density lipoprotein cholesterol (HDL-C), and low-density lipoprotein cholesterol (LDL-C). Renal function indicators included serum creatinine, estimated glomerular filtration rate, and urinary albumin/creatinine ratio. Liver function indicators included alanine aminotransferase and aspartate aminotransferase.

### Monitoring procedure

A standard 12-lead 24-hour ambulatory electrocardiographic recorder was used for HRV assessment. Monitoring was performed within 1 week before initiation of GLP-1 RA therapy and again within 1 week after 12 weeks of treatment.

To improve consistency and minimize circadian variation, Holter recordings were initiated between 8:00 and 10:00 AM. Patients were instructed to maintain their usual sleep cycles and medication schedules during recording periods. They were also asked to continue their usual daily activities while avoiding intensive exercise and emotional stress. Each participant kept an activity log documenting routine activities, symptoms, and medication timing.

### HRV parameters

Time-domain HRV parameters included SDNN, defined as the standard deviation of all normal-to-normal RR intervals over 24 hours; SDANN, defined as the standard deviation of the average normal RR intervals for each 5-minute segment over 24 hours; RMSSD, defined as the root mean square of successive differences between adjacent normal RR intervals; and pNN50, defined as the percentage of adjacent normal RR interval differences >50 ms.

Frequency-domain HRV parameters included low-frequency power (LF), high-frequency power (HF), and the LF/HF ratio. LF reflects combined sympathetic and parasympathetic modulation, whereas HF reflects parasympathetic activity. The LF/HF ratio was used as an index of sympathovagal balance, while acknowledging that its physiological interpretation remains debated. For statistical analysis, logarithmically transformed LF and HF values (lnLF and lnHF) were used, and the logarithmically expressed LF/HF ratio was also analyzed.

Heart rate parameters included maximum, minimum, and mean heart rate over 24 hours.

The primary outcome was the change in SDNN from baseline to 12 weeks. Changes in SDANN, RMSSD, pNN50, lnLF, lnHF, and lnLF/lnHF were analyzed as secondary exploratory outcomes.

### Statistical analysis

**Figure 1:**
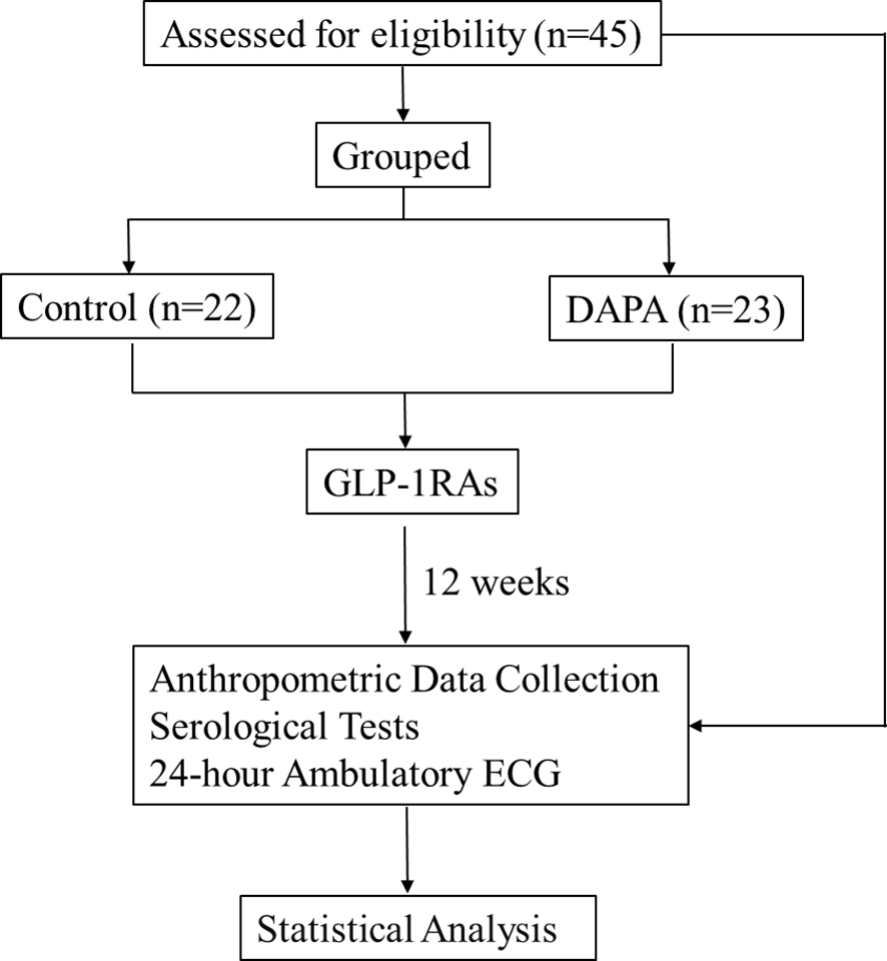
Study flow diagram.

Continuous variables were summarized as mean ± standard deviation or median [Q1, Q3], and categorical variables as number (percentage). Baseline characteristics were compared using the independent-samples t test or Mann–Whitney U test for continuous variables and the chi-square test or Fisher’s exact test for categorical variables, with absolute standardized mean differences (SMDs) additionally calculated to assess baseline balance. Within-group comparisons between baseline and 12 weeks were performed using paired t tests or Wilcoxon signed-rank tests, and unadjusted between-group comparisons of change scores (Δ values) were performed using the independent-samples t test or Mann–Whitney U test. Adjusted between-group comparisons for HRV change outcomes were conducted using analysis of covariance (ANCOVA), including treatment group as the fixed factor and the corresponding baseline HRV value, age, sex, diabetes duration, body mass index (BMI), HbA1c, HOMA-B, and HDL-C as covariates; adjusted estimates were reported as least-squares mean changes with 95% confidence intervals. As a sensitivity analysis, inverse probability of treatment weighting (IPTW) based on propensity scores was performed using age, sex, diabetes duration, BMI, HbA1c, HOMA-B, and HDL-C, with balance assessed by absolute SMDs and weighted outcome models additionally adjusted for the corresponding baseline HRV value. Multivariable linear regression models were also fitted for each HRV change outcome with the same covariate set; for presentation, the coefficients for baseline dapagliflozin use and the corresponding baseline HRV value were emphasized. All tests were two-sided, and p<0.05 was considered statistically significant. All analyses were performed using R. The flowchart of the study is shown in **Figure 1**.

### Ethical approval

This study was approved by the Ethics Committee of Nanjing First Hospital. All procedures were conducted in accordance with the Helsinki Declaration of 1964, as revised in 2013. Written informed consent was obtained from all participants. The study was registered at (NCT05611684).

## Results

### Baseline characteristics

A total of 45 patients with T2DM were included in this study, including 22 patients in the Control group and 23 in the DAPA group. Baseline characteristics according to baseline dapagliflozin use are presented in **Table 1**. Overall, the two groups were broadly comparable in terms of clinical characteristics, medical history, and concomitant medications. However, modest between-group imbalances were observed for several baseline metabolic variables, including diabetes duration, triglycerides, and HDL-C. Therefore, in addition to unadjusted comparisons, absolute standardized mean differences were examined, and subsequent adjusted analyses were performed to account for potential baseline confounding. The distribution of GLP-1 RA types was comparable between groups. In the Control group, 10 patients received polyethylene glycol loxenatide and 12 received semaglutide, whereas in the DAPA group, 11 received polyethylene glycol loxenatide and 12 received semaglutide.

**Table 1:** Baseline characteristics of the study population.

Variable	Control (n = 22)	DAPA (n = 23)	SMD	P value
Clinical characteristics				
Age, years	58.73 ± 10.26	54.13 ± 8.79	0.481	0.115
Male sex, n (%)	16 (72.7%)	13 (56.5%)	0.344	0.256
Duration, years	5.00 [3.00, 7.00]	2.00 [1.00, 4.50]	0.580	0.025
Body weight, kg	72.25 [69.00, 78.88]	72.80 [66.30, 79.50]	0.061	0.991
BMI, kg/m²	25.44 [24.61, 27.09]	27.30 [24.87, 28.53]	0.442	0.125
Waist circumference, cm	95.54 ± 9.26	95.59 ± 7.57	0.006	0.984
Hip circumference, cm	99.85 ± 7.27	101.62 ± 6.19	0.261	0.387
Waist-to-Hip Ratio	0.96 ± 0.05	0.94 ± 0.04	0.358	0.238
SBP, mmHg	130.50 [118.50, 140.50]	124.00 [118.50, 131.00]	0.487	0.215
DBP, mmHg	81.00 [75.50, 90.00]	83.00 [76.50, 88.00]	0.197	0.937
Medical history				
Hypertension, n (%)	12 (54.5%)	11 (47.8%)	0.134	0.652
Cardiovascular disease, n (%)	8 (36.4%)	10 (43.5%)	0.145	0.626
Stroke, n (%)	7 (31.8%)	10 (43.5%)	0.241	0.420
Diabetic nephropathy, n (%)	9 (40.9%)	10 (43.5%)	0.052	0.862
Diabetic retinopathy, n (%)	9 (40.9%)	10 (43.5%)	0.052	0.862
Diabetic peripheral neuropathy, n (%)	11 (50.0%)	11 (47.8%)	0.043	0.884
Medical therapy				
Metformin use, n (%)	8 (36.4%)	5 (21.7%)	0.322	0.279
Insulin use, n (%)	2 (9.1%)	4 (17.4%)	0.245	0.665
Sulfonylurea use, n (%)	5 (22.7%)	6 (26.1%)	0.078	0.793
Statin use, n (%)	8 (36.4%)	5 (21.7%)	0.322	0.279
Antiplatelet use, n (%)	5 (22.7%)	4 (17.4%)	0.133	0.722
Metabolic Index				
HbA1c, %	8.60 [8.20, 9.40]	8.20 [7.40, 9.10]	0.126	0.182
Fasting blood glucose, mmol/L	9.16 ± 1.78	8.05 ± 3.35	0.415	0.188
Fasting insulin, mU/L	5.24 [4.52, 8.78]	8.30 [5.00, 16.64]	0.592	0.074
Fasting glucagon, pmol/L	12.30 [10.79, 15.00]	11.91 [10.35, 13.65]	0.076	0.496
Fasting C-peptide, ng/mL	1.58 [1.36, 2.22]	2.35 [1.34, 3.00]	0.470	0.274
TyG index	9.24 ± 0.48	9.27 ± 0.79	0.057	0.855
HOMA-IR	2.39 [1.73, 3.92]	3.15 [2.40, 5.96]	0.644	0.118
HOMA-IS	15.06 [11.91, 17.35]	15.10 [12.79, 21.38]	0.441	0.503
HOMA-B	5.24 [2.95, 8.97]	8.79 [4.34, 13.64]	0.486	0.162
Total cholesterol, mmol/L	4.80 ± 1.19	4.70 ± 1.05	0.093	0.766
Triglycerides, mmol/L	1.46 [1.05, 1.91]	1.96 [1.13, 2.59]	0.631	0.080
HDL-C, mmol/L	1.24 ± 0.27	1.02 ± 0.30	0.780	0.016
LDL-C, mmol/L	3.01 ± 0.98	2.93 ± 1.04	0.078	0.802
Lpa, ng/L	76.00 [53.00, 123.00]	73.00 [40.00, 190.00]	0.122	0.900

Values are presented as mean ± SD, median [Q1, Q3], or n (%). P values were calculated using the t test or Mann–Whitney U test for continuous variables, and the chi-square test or Fisher’s exact test for categorical variables. Absolute standardized mean differences (SMDs) are additionally presented to aid assessment of baseline balance between groups. Abbreviations: BMI, body mass index; DBP, diastolic blood pressure; DAPA, dapagliflozin; HbA1c, glycated hemoglobin; HDL-C, high-density lipoprotein cholesterol; HOMA-B, homeostasis model assessment of β-cell function; HOMA-IR, homeostasis model assessment of insulin resistance; HOMA-IS, homeostasis model assessment of insulin sensitivity; LDL-C, low-density lipoprotein cholesterol; Lpa, lipoprotein(a); SBP, systolic blood pressure; TyG, triglyceride-glucose.

### Within group changes in heart rate and heart rate variability

**Figure 2:**
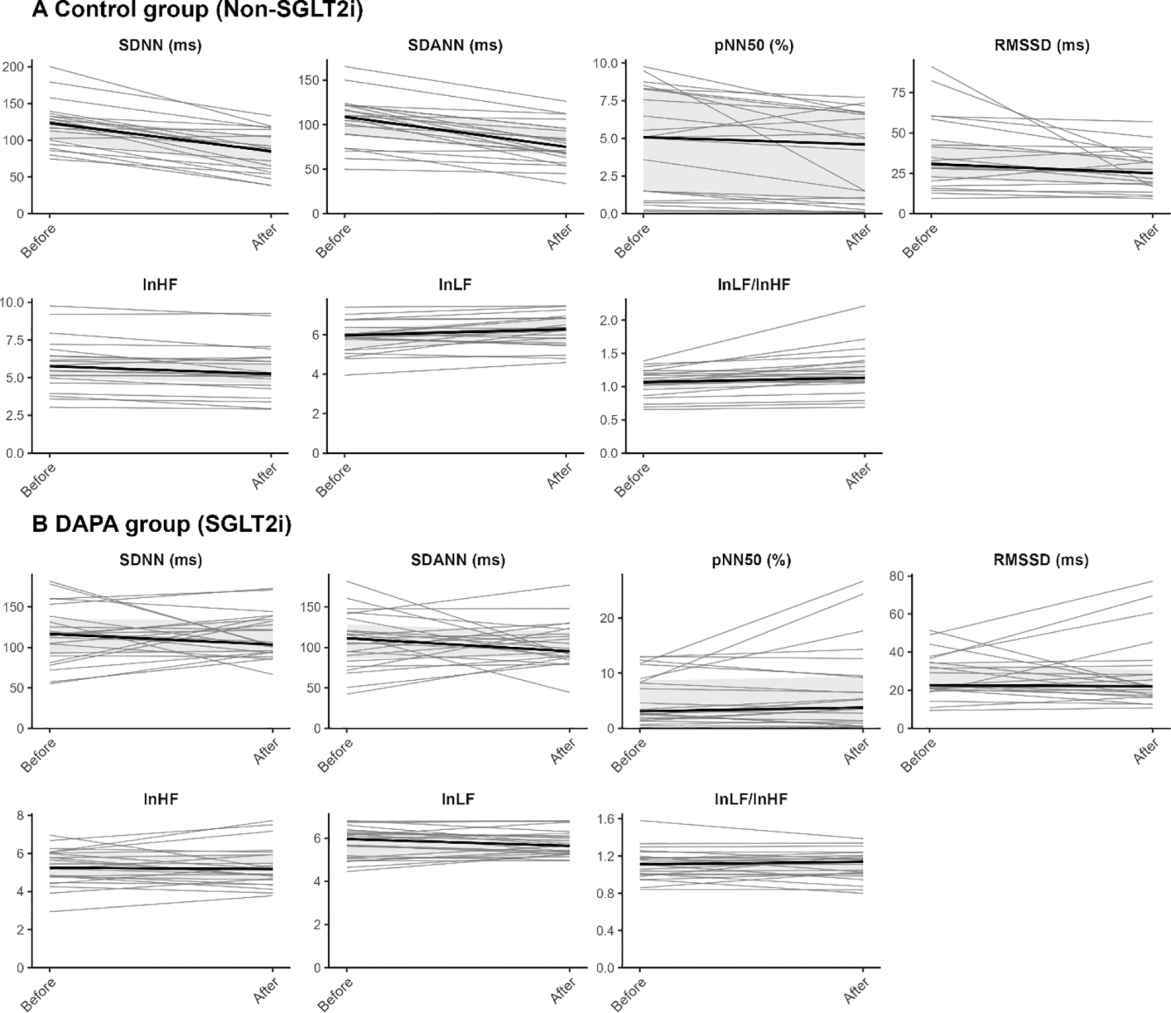
Individual trajectories of HRV parameters before and after 12 weeks of GLP-1 receptor agonist therapy by group. Panels **(A, B)** show within-group changes in HRV parameters in the Control group and DAPA group, respectively. Gray lines represent individual participant trajectories from baseline to 12 weeks, and black lines indicate the overall group-level trend.

Within-group changes in HR and HRV after 12 weeks of GLP-1 receptor agonist therapy are summarized in **Table 2**, **Figure 2**. In the Control group, no significant changes were observed in maximum HR, minimum HR, or mean HR (all p>0.05). However, SDNN and SDANN decreased significantly from baseline (both p<0.001), and RMSSD and pNN50 also declined (both p=0.003). In the frequency domain, lnLF increased (p=0.011), whereas lnHF decreased (p=0.001). The lnLF/lnHF ratio also increased significantly (p<0.001). In the DAPA group, no significant within-group changes were observed in HR parameters or HRV indices over the 12-week follow-up (all p>0.05).

**Table 2:** Within-group changes in HR and HRV after 12 weeks of GLP-1 receptor agonist therapy.

Parameter	Control (n = 22)		P value	DAPA (n = 23)		P value
	Before	After		Before	After	
HR parameters (bpm)						
Maximum HR (bpm)	121.23 ± 15.50	121.27 ± 14.31	0.987	120.70 ± 13.30	117.39 ± 15.51	0.266
Minimum HR (bpm)	54.00 [51.00, 60.00]	56.50 [51.75, 69.00]	0.064	56.00 [53.50, 64.50]	63.00 [56.00, 67.00]	0.394
Mean HR (bpm)	80.00 [73.50, 81.99]	83.00 [75.00, 88.27]	0.205	77.78 [70.68, 83.19]	82.00 [75.50, 87.40]	0.300
24-h time domain						
SDNN (ms)	121.87 ± 30.65	84.14 ± 27.61	<0.001	115.79 ± 35.46	114.12 ± 27.55	0.831
SDANN (ms)	104.51 ± 27.72	78.21 ± 23.61	<0.001	108.45 ± 34.63	103.60 ± 27.49	0.550
RMSSD (ms)	30.84 [20.84, 45.00]	25.09 [18.06, 34.04]	0.003	22.62 [20.68, 34.48]	22.10 [17.35, 30.57]	0.707
pNN50 (%)	5.08 [1.50, 8.28]	4.60 [0.76, 6.62]	0.003	3.10 [1.75, 8.66]	3.77 [1.37, 9.37]	0.560
24-h Frequency domain						
lnLF	5.89 ± 0.81	6.19 ± 0.82	0.011	5.78 ± 0.73	5.73 ± 0.57	0.643
lnHF	5.76 [5.01, 6.45]	5.25 [4.59, 6.26]	0.001	5.25 [4.78, 6.01]	5.18 [4.65, 5.94]	0.849
lnLF/lnHF	1.07 [0.97, 1.20]	1.14 [1.07, 1.35]	<0.001	1.11 [1.01, 1.19]	1.14 [1.01, 1.20]	0.635

Values are presented as mean ± SD or median [Q1, Q3]. P values represent within-group comparisons between baseline and 12 weeks using paired t tests or Wilcoxon signed-rank tests. Abbreviations: DAPA, dapagliflozin; HF, high frequency; HR, heart rate; LF, low frequency; RMSSD, root mean square of successive RR interval differences; SDANN, standard deviation of the average normal-to-normal intervals for each 5-minute segment; SDNN, standard deviation of all normal-to-normal intervals.

### Between group differences in changes in heart rate and heart rate variability

**Figure 3:**
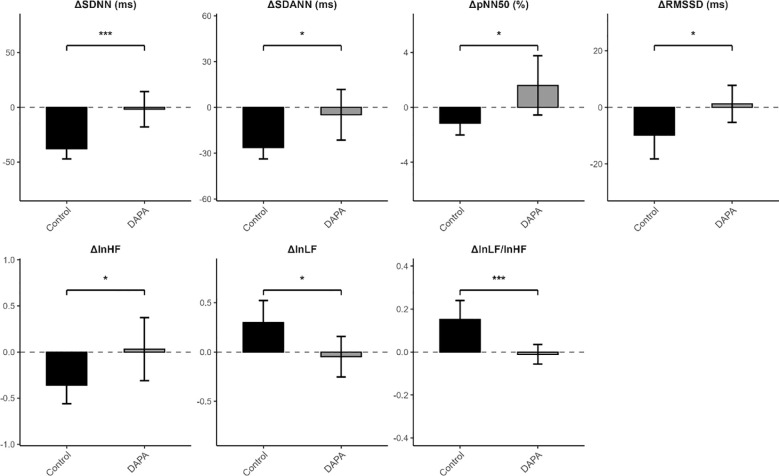
Between-group differences in changes in HRV parameters after 12 weeks of GLP-1 receptor agonist therapy. Bars represent mean change and error bars indicate standard errors. Negative values indicate a decrease from baseline, whereas positive values indicate an increase. *p<0.05; ***p<0.001.

Unadjusted between-group comparisons of change in HR and HRV are presented in **Table 3**, **Figure 3**. Changes in maximum HR, minimum HR, and mean HR, did not differ significantly between the Control and DAPA groups (all p>0.05). In contrast, significant differences between two groups were observed for HRV parameters. Compared with the Control group, the DAPA group showed a decline in SDNN (−1.67 ± 37.27 vs. −37.73 ± 21.23, p<0.001) and SDANN (−4.85 ± 38.28 vs. −26.30 ± 16.89, p=0.020). Similarly, RMSSD and pNN50 were better preserved in the DAPA group than in the Control group. In the frequency domain, the DAPA group showed a reduction in lnHF, whereas lnLF decreased slightly in the DAPA group but increased in the Control group. In addition, the change in the lnLF/lnHF ratio differed significantly between groups.

**Table 3:** Unadjusted between-group differences in changes in HR and HRV.

Outcome	Control (n = 22)	DAPA (n = 23)	SMD	P value
Δ Maximum HR (bpm)	0.05 ± 13.33	-3.30 ± 13.89	-0.246	0.414
Δ Minimum HR (bpm)	3.59 ± 8.61	0.78 ± 11.05	-0.284	0.750
Δ Mean HR (bpm)	2.31 ± 8.27	2.18 ± 9.86	-0.013	0.964
Δ SDNN (ms)	-37.73 ± 21.23	-1.67 ± 37.27	1.189	<0.001
Δ SDANN (ms)	-26.30 ± 16.89	-4.85 ± 38.28	0.725	0.020
Δ RMSSD (ms)	-9.79 ± 19.03	1.20 ± 15.15	0.639	0.048
Δ pNN50 (%)	-1.14 ± 1.97	1.60 ± 4.99	0.723	0.038
Δ lnLF	0.30 ± 0.50	-0.05 ± 0.47	-0.709	0.022
Δ lnHF	-0.36 ± 0.45	0.03 ± 0.79	0.608	0.048
Δ lnLF/lnHF	0.15 ± 0.20	-0.01 ± 0.10	-1.016	<0.001

Δ values represent follow-up minus baseline. Values are presented as mean ± SD. P values were derived from unadjusted between-group comparisons of change scores using the t test or Mann–Whitney U test. DAPA, dapagliflozin; HR, heart rate; HRV, heart rate variability; SMD, standardized mean difference.

### Adjusted between-group comparisons using ANCOVA

**Figure 4:**
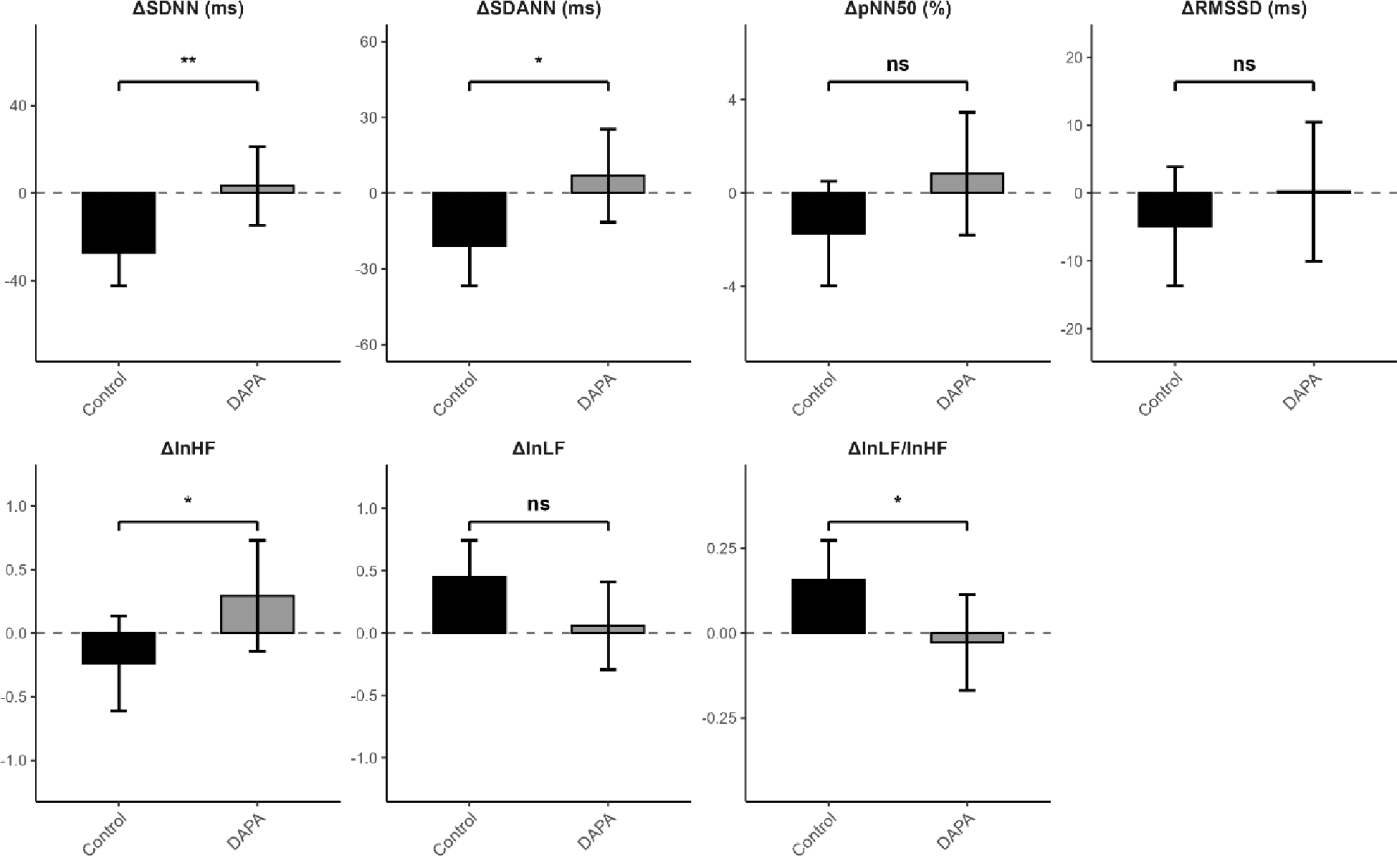
Adjusted between-group differences in HRV changes based on ANCOVA models *p<0.05; **p<0.01; ns, not significant.

To account for potential baseline confounding, analysis of covariance (ANCOVA) was performed for changes in HRV parameters. Adjusted comparisons are presented in **Table 4**, **Figure 4**. The ANCOVA models included baseline HRV value, age, sex, diabetes duration, BMI, HbA1c, HOMA-B, and HDL-C as covariates. After adjustment, significant between-group differences remained for HRV parameters. Compared with the Control group, the DAPA group showed more favorable adjusted changes in SDNN (p=0.005), SDANN (p=0.011), lnHF (p=0.036), and the lnLF/lnHF ratio (p=0.026). By contrast, adjusted between-group differences were not statistically significant for RMSSD (p=0.368), pNN50 (p=0.086), or lnLF (p=0.059). These adjusted analyses indicate that the between-group differences in several HRV parameters persisted after accounting for selected baseline covariates.

**Table 4:** Adjusted between-group differences in changes in HRV.

Outcome	Control (95% CI)	DAPA (95% CI)	Partial η²	P value
Δ SDNN (ms)	-27.31 (-42.34 to -12.28)	3.33 (-14.56 to 21.21)	0.316	0.005
Δ SDANN (ms)	-21.03 (-36.77 to -5.29)	6.91 (-11.50 to 25.33)	0.269	0.011
Δ RMSSD (ms)	-4.91 (-13.67 to 3.85)	0.23 (-10.01 to 10.47)	0.039	0.368
Δ pNN50 (%)	-1.74 (-3.97 to 0.50)	0.83 (-1.80 to 3.46)	0.134	0.086
Δ lnLF	0.45 (0.16 to 0.74)	0.06 (-0.29 to 0.41)	0.160	0.059
Δ lnHF	-0.24 (-0.61 to 0.13)	0.29 (-0.14 to 0.73)	0.193	0.036
Δ lnLF/lnHF	0.16 (0.04 to 0.27)	-0.03 (-0.17 to 0.11)	0.214	0.026

Each model included treatment group, the corresponding baseline HRV value, age, sex, diabetes duration, BMI, HbA1c, HOMA-B, and HDL-C. Adjusted estimates are model-based least-squares mean changes with 95% confidence intervals. P values correspond to the adjusted between-group contrast. Partial η² is presented as a measure of effect size.

### IPTW-weighted sensitivity analysis

To further assess the robustness of the primary findings, an IPTW-weighted sensitivity analysis was performed. Baseline covariate balance improved substantially after weighing (**Table 5**). The absolute standardized mean differences were reduced for all included covariates after weighting, with all weighted SMDs ≤0.126, indicating acceptable balance between groups.

**Table 5:** Baseline covariate balance before and after IPTW.

Covariate	Unweighted SMD	Weighted SMD
Age, years	0.481	0.028
Male sex	0.339	0.094
Diabetes duration, years	0.580	0.052
BMI, kg/m²	0.442	0.028
HbA1c, %	0.129	0.126
HOMA-B	0.207	0.084
HDL-C, mmol/L	0.755	0.059

Baseline covariate balance was assessed using absolute standardized mean differences (SMDs) before and after inverse probability of treatment weighting (IPTW). Lower SMD values after weighting indicate improved balance between groups.

**Figure 5:**
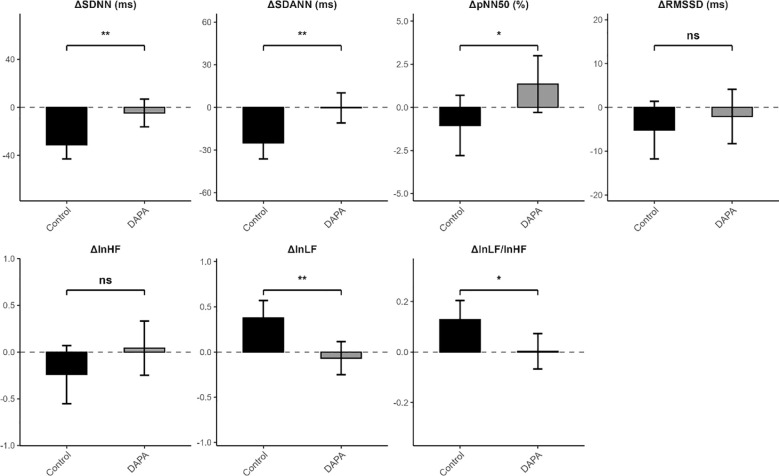
IPTW-weighted adjusted between-group differences in HRV changes. Bars represent IPTW-weight adjusted mean changes, and error bars indicate 95% confidence intervals. The propensity score model included age, sex, diabetes duration, BMI, HbA1c, HOMA-B, and HDL-C. *p<0.05; **p<0.01; ns, not significant.

The IPTW-weight adjusted comparisons of HRV changes are presented in **Table 6**, **Figure 5**. After weighing, significant between-group differences remained for SDNN (p=0.002), SDANN (p=0.002), pNN50 (p=0.040), lnLF (p=0.001), and the lnLF/lnHF ratio (p=0.013). By contrast, the weighted between-group differences were not statistically significant for RMSSD (p=0.478) or lnHF (p=0.171). The IPTW-weighted sensitivity analysis yielded results consistent with the primary adjusted analyses, supporting the robustness of the observed between-group differences in several HRV parameters.

**Table 6:** IPTW-weighted adjusted between-group differences in HRV changes.

Outcome	Control (95% CI)	DAPA (95% CI)	Partial η²	P value
Δ SDNN (ms)	-31.12 (-42.95 to -19.29)	-4.67 (-16.14 to 6.81)	0.250	0.002
Δ SDANN (ms)	-25.02 (-36.30 to -13.74)	-0.36 (-10.89 to 10.17)	0.252	0.002
Δ RMSSD (ms)	-5.18 (-11.78 to 1.41)	-2.10 (-8.31 to 4.11)	0.014	0.478
Δ pNN50 (%)	-1.05 (-2.80 to 0.70)	1.35 (-0.29 to 2.99)	0.115	0.040
Δ lnLF	0.38 (0.19 to 0.57)	-0.07 (-0.25 to 0.11)	0.258	0.001
Δ lnHF	-0.24 (-0.55 to 0.07)	0.04 (-0.25 to 0.33)	0.053	0.171
Δ lnLF/lnHF	0.13 (0.05 to 0.20)	0.00 (-0.07 to 0.07)	0.163	0.013

IPTW-weighted adjusted comparisons were performed to evaluate between-group differences in changes in HRV parameters. The propensity score model included age, sex, diabetes duration, BMI, HbA1c, HOMA-B, and HDL-C. Weighted outcome models additionally adjusted for the corresponding baseline HRV value. Adjusted estimates are weighted least-squares mean changes with 95% confidence intervals. P values correspond to weighted between-group contrasts. Partial η² is presented as a measure of effect size. CI, confidence interval; DAPA, dapagliflozin; HDL-C, high-density lipoprotein cholesterol; HRV, heart rate variability; IPTW, inverse probability of treatment weighting.

### Multivariable linear regression analysis of factors associated with changes in heart rate variability

To further explore factors associated with changes in HRV, multivariable linear regression models were fitted for each ΔHRV outcome. For each model, DAPA and the corresponding baseline HRV value are presented in **Table 7**, while all models were adjusted for age, sex, diabetes duration, BMI, HbA1c, HOMA-B, and HDL-C. In these exploratory analyses, baseline DAPA use was significantly associated with a more favorable change in SDNN (estimate 30.634, 95% CI 1.472 to 59.795; p=0.040). A similar direction of association was observed for SDANN, although this did not reach statistical significance (estimate 27.939, 95% CI −0.237 to 56.114; p=0.052). For RMSSD, pNN50, lnLF, lnHF, and the lnLF/lnHF ratio, the coefficients for DAPA were not statistically significant.

**Table 7:** Multivariable linear regression models for changes in HRV.

Dependent variable	Independent variable	Estimate (95% CI)	P value	R²	Adjusted R²
Δ SDNN (ms)	DAPA	30.634 (1.472 to 59.795)	0.040	0.569	0.385
	Baseline value	-0.613 (-1.067 to -0.159)	0.011		
Δ SDANN (ms)	DAPA	27.939 (-0.237 to 56.114)	0.052	0.499	0.284
	Baseline value	-0.578 (-1.138 to -0.017)	0.044		
Δ RMSSD (ms)	DAPA	5.141 (-9.989 to 20.272)	0.488	0.427	0.181
	Baseline value	-0.505 (-1.195 to 0.185)	0.143		
Δ pNN50 (%)	DAPA	2.568 (-1.207 to 6.344)	0.172	0.270	-0.042
	Baseline value	-0.075 (-0.591 to 0.442)	0.767		
Δ lnLF	DAPA	-0.392 (-0.905 to 0.122)	0.128	0.402	0.146
	Baseline value	-0.253 (-0.622 to 0.116)	0.169		
Δ lnHF	DAPA	0.533 (-0.158 to 1.223)	0.123	0.354	0.077
	Baseline value	-0.062 (-0.304 to 0.180)	0.598		
Δ lnLF/lnHF	DAPA	-0.184 (-0.403 to 0.034)	0.094	0.339	0.056
	Baseline value	0.224 (-0.562 to 1.010)	0.560		

Only the coefficients for DAPA and the corresponding baseline outcome are shown for each model. All models were adjusted for age, sex, diabetes duration, BMI, HbA1c, HOMA-B, and HDL-C. Estimates are unstandardized regression coefficients with 95% confidence intervals for Δ outcomes (follow-up minus baseline). R² and adjusted R² are reported for the full models. Abbreviations: CI, confidence interval; DAPA, dapagliflozin; HDL-C, high-density lipoprotein cholesterol; HRV, heart rate variability.

The corresponding baseline HRV value was inversely associated with changes in SDNN (estimate −0.613, 95% CI −1.067 to −0.159; p=0.011) and SDANN (estimate −0.578, 95% CI −1.138 to −0.017; p=0.044), indicating that higher baseline values were associated with larger subsequent declines in these measures. No significant associations were observed between baseline HRV values and changes in the other HRV parameters. The full coefficient estimates for all multivariable regression models are provided in **Supplementary Table 1**.

## Discussion

This study is the first to explore the potential moderating effect of SGLT2i (dapagliflozin) on cardiac autonomic function changes in patients with T2DM initiated on GLP-1 RAs in clinical practice. A total of 45 patients with T2DM were included in this study. The results showed that, after three months of GLP-1 RA treatment, multiple HRV indices deteriorated in the control group, including time-domain measures such as SDNN, SDANN, RMSSD, and pNN50, together with changes in frequency-domain indices including lnLF, lnHF, and the lnLF/lnHF ratio. In contrast, among patients whose baseline regimen included dapagliflozin, GLP-1 RA therapy was not associated with significant within-group worsening of HRV indices, and HRV measures remained relatively stable over time. In unadjusted between-group comparisons, significant differences were observed for several HRV parameters. After adjustment using ANCOVA, between-group differences remained significant for SDNN, SDANN, lnHF, and the lnLF/lnHF ratio. Moreover, IPTW-weighted sensitivity analyses yielded consistent findings, with significant between-group differences persisting for SDNN, SDANN, pNN50, lnLF, and the lnLF/lnHF ratio. In the multivariable linear regression analyses, baseline dapagliflozin use was most clearly associated with a more favorable change in SDNN, whereas evidence for the other HRV outcomes was weaker. These findings suggest that baseline dapagliflozin use was associated with attenuation of GLP-1 RA-associated HRV decline, particularly for indices reflecting overall variability, although this observation should be interpreted cautiously given the observational nature of the study. This finding provides new insights for optimizing combination therapy in patients with T2DM.

Diabetic autonomic neuropathy is defined as a heterogeneous category of disorders of the autonomic nervous system (ANS) in individuals with either diabetes or metabolic derangements of pre-diabetes, when other potential causes have been excluded (9). Currently, non-invasive methods for assessing cardiac autonomic function primarily rely on HRV. HRV effectively reflects autonomic modulation of cardiac rhythm by analyzing various metrics of interbeat intervals such as SDNN, SDANN, RMSSD, pNN50, LF, HF, and LF/HF (lnLF/lnHF). Specifically, SDANN, RMSSD, pNN50, and HF all reflect cardiac parasympathetic nerve activity, while the LF/HF ratio is commonly used to evaluate the relative balance of sympathetic and parasympathetic activity, although its interpretation as a direct index of sympathetic activity remains debated (3, 8). Despite the well-recognized cardiovascular benefits of GLP-1 RAs in T2DM (10–12), a substantial body of clinical and experimental evidence has documented their effects on heart rate and HRV. It is unclear how GLP-1 RAs affect the autonomic nervous system, and these effects may be more complex than mechanisms related to peripheral neuropathy because of the many inputs that determine both sympathetic and parasympathetic autonomic activities (13).

Furthermore, the role of GLP-1 RAs on sympathetic drive is particularly debated. For example, GLP-1RAs have been observed in the carotid body and activation of these diminish the sympathetic response to high plasma glucose and/or insulin (14). Conversely, GLP-1RAs cause an increased resting heart rate (increased by approximately 3 beats-per-min), which may suggest an increase in sympathetic drive (13). Other studies have reported decreased vagal tone, decreased 30:15 value from the lying-to-standing test, and a decreased heart rate variability with GLP-1 RAs (13, 15).

All this evidence indicates that the effects of GLP-1 RAs on the sympathetic and parasympathetic nervous systems are multifaceted, involving both direct and indirect mechanisms. However, this phenomenon requires further validation through well-designed clinical and experimental studies.

In this study, GLP-1 RA treatment was associated with no statistically significant change in maximum, minimum, or mean heart rate within either group over 12 weeks. However, in the control group, several HRV parameters worsened significantly over time. SDNN and SDANN, indices reflecting overall and longer-term variability, decreased significantly, while RMSSD and pNN50, which are more sensitive to parasympathetic modulation, also declined. In the frequency domain, lnHF decreased, whereas lnLF and the lnLF/lnHF ratio increased. By contrast, no significant within-group changes in HRV indices were observed in the dapagliflozin group. In the unadjusted between-group analysis, patients in the dapagliflozin group showed more favorable changes in SDNN, SDANN, RMSSD, pNN50, lnLF, lnHF, and lnLF/lnHF. After covariate adjustment, significant between-group differences remained for SDNN, SDANN, lnHF, and lnLF/lnHF, and the IPTW-weighted sensitivity analysis supported the robustness of several of these findings. The reduction in SDNN and SDANN in the control group generally indicates reduced overall adaptability of the heart to internal and external environmental changes, which has been linked to cardiovascular risk. However, the observed reduction in SDNN in the control group, while statistically significant, still requires further study to determine its clinical relevance in terms of cardiovascular risk prediction in this specific treatment context. RMSSD and pNN50 are sensitive indicators reflecting the instantaneous regulatory capacity of the vagus nerve (parasympathetic) on the heart, and their reduction is compatible with reduced vagal modulation. Simultaneously, changes in lnLF, lnHF, and lnLF/lnHF are suggestive of altered sympathovagal balance, although these frequency-domain measures should be interpreted with caution, particularly the LF/HF ratio, which remains a debated marker (8).

This series of changes suggests that GLP-1 RA treatment may reduce overall heart rate variability and alter cardiac autonomic balance in some patients, particularly in those without baseline dapagliflozin use. The primary findings of this study are generally consistent with previous research. For example, Kumarathurai et al. (16) reported detrimental reductions in multiple HRV indicators in patients with T2DM after 12 weeks of liraglutide treatment. Specifically, liraglutide reduced SDNN, RMSSD, and HF, whereas the LF/HF ratio showed no change compared with the placebo (16). Berkelaar et al. (17) conducted a study involving 130 healthy subjects and found that GLP-1RAs induce a transient increase in heart rate, but cardiac vagal function was not significantly affected. The study also demonstrated a positive correlation between serum insulin levels and heart rate (17), suggesting that the effects of GLP-1RAs may be mediated through an increase in insulin levels (18). Other experiments have confirmed that GLP-1RAs activate the sympathetic nervous system in healthy individuals (19, 20). In animal models, GLP-1 binds to GLP-1RAs in the central and peripheral nervous systems as well as in the autonomic nervous system (ANS), thereby enhancing sympathetic nervous system activity and reducing parasympathetic nervous system activity (21). In this regard, Baggio et al. (21) proposed that the GLP-1RA-associated increase in heart rate is the final effect of a direct chronotropic action, which can be attenuated by propofol but not by atropine. Furthermore, GLP-1RAs increase c-fos expression (a marker of neuronal activity) in the adrenal medulla, activate neurons involved in central autonomic control, and stimulate tyrosine hydroxylase transcription in brainstem catecholamine neurons (22). These findings suggest that central GLP-1R actions may contribute to the modulation of sympathetic pathways (22). However, some studies have reported divergent results. In a recent meta-analysis, Greco et al. confirmed an increase in heart rate but found no alteration in sympatho-vagal balance with chronic use of GLP-1RAs in individuals with diabetes (15). Jaiswal et al. reported no superiority in terms of CARTs or HRV following treatment with exenatide compared with insulin over 18 months in a small randomised trial (n=46) (23). Recently, an observational study over 12 weeks found that GLP-1RAs (semaglutide/dulaglutide) improved nerve size and nerve morphology, reduced the severity of neuropathy, and improved sural sensory nerve conduction amplitude, suggesting a direct structural improvement to the nervous system (24). While evidence of the direct impact of GLP-1RAs on CAN is limited, in light of the limited amount of available data, the actual impact of long-term GLP-1RA use on the autonomic nervous system should be interpreted with caution.

As a novel class of glucose-lowering agents, dapagliflozin has demonstrated positive effects on cardiovascular health in multiple cardiovascular outcome trials, extending beyond glycemic benefits. Studies have shown that dapagliflozin improves cardiac autonomic function indicators (6), and reduces the recurrence of vasovagal syncope in patients with T2DM (7). In the present study, patients whose background medication included dapagliflozin maintained relatively stable HRV indicators after 12 weeks of GLP-1 RA treatment. In addition, the between-group differences observed in the primary analyses persisted in several key HRV parameters after ANCOVA adjustment and remained broadly consistent in IPTW-weighted sensitivity analyses. Although the exploratory multivariable regression models suggested that the association of dapagliflozin was most evident for SDNN, the overall pattern across the adjusted and weighted analyses supports the possibility that baseline dapagliflozin use may be associated with preservation of autonomic stability during GLP-1 RA therapy.

Although direct evidence on HRV is still limited, available data suggest that time-domain parameters such as SDNN and RMSSD may be elevated in patients on SGLT2i (25). In the frequency domain, preliminary results of a decrease in the lnLF/lnHF ratio and an increase in HF power may be interpreted as a decrease in sympathetic dominance and/or an increase in vagal influence (26). This finding aligns with a recent review, which explicitly states that SGLT2i can improve HRV by attenuating sympathetic over-activity and promoting parasympathetic regulation, specifically manifested as increases in parameters such as SDNN and RMSSD and a decrease in the LF/HF ratio (8). The potential attenuation of sympathetic nervous activity with SGLT2i has been hypothesized to involve multiple mechanisms, including reductions in circulating insulin levels (27), weight loss (28), and improved baroreceptor sensitivity (29). Improved glycemic control reduces hyperglycemia-induced elevation in serum insulin levels, collectively contributing to a more balanced autonomic state. Furthermore, concomitant weight loss and decreased leptin concentrations contribute to a reduction in sympathetic tone (7). Additionally, SGLT2 inhibitors exert beneficial effects on cardiovascular regulation through modulation of baroreceptor sensitivity (30) and attenuation of oxidative stress and inflammatory processes (31, 32). SGLT2 inhibitors, through osmotic diuresis, sodium excretion, and mild blood volume contraction, lower blood pressure and reduce cardiac preload. Concurrently, the mild blood volume changes they induce may be mediated by baroreflex mechanisms, suppressing sympathetic nerve output and enhancing parasympathetic activity (8). SGLT2 inhibitors may improve cardiac autonomic nerve function through multiple mechanisms (metabolic, hemodynamic, anti-inflammatory, etc.), specifically by regulating HRV, thereby reducing sympathetic nerve activity and enhancing parasympathetic nerve regulation. This may be one of the important mechanisms underlying their cardiovascular protection, especially in reducing the risk of heart failure hospitalization (8). This complementary hemodynamic effect may directly counteract the potential sympathetic excitation tendency that could be triggered by GLP-1RA. Collectively, these multifaceted mechanisms underscore the potential of SGLT2i to restore autonomic balance and improve cardiovascular outcomes in patients with T2DM. Inflammation (33) and oxidative stress (34) are key molecular mechanisms contributing to the development and progression of CAN. The combined use of SGLT-2i and GLP-1RA can synergistically reduce the levels of inflammatory and oxidative stress biomarkers (35, 36), which likely forms an important foundation for their ability to improve cardiac electrophysiological stability and autonomic nervous function. The above hypothesis requires further research for confirmation. The interplay between SGLT2 inhibition and autonomic function remains an active area of investigation (37).

In summary, combination therapy with GLP-1 RAs and SGLT2i has become a preferred treatment strategy for glycemic and weight control, while also providing cardiorenal protective benefits. Our study suggests that such a combination may not only confer advantages in metabolic outcomes and hard endpoints but may also help preserve cardiac autonomic function, an important intermediate marker, in some patients. For patients with T2DM who are planned to initiate GLP-1 RA therapy, particularly those already at risk of early cardiac autonomic neuropathy (e.g., elevated resting heart rate, borderline reduction in heart rate variability), incorporating an SGLT2i (such as dapagliflozin) in advance or as part of the combination regimen may be a strategy associated with better preservation of cardiac autonomic stability. However, this clinical implication should be interpreted cautiously and requires confirmation in future prospective studies.

It is crucial to emphasize that this study investigated mechanistic surrogate markers (HRV parameters) of cardiac autonomic function. We did not assess hard clinical outcomes such as arrhythmic events, hospitalizations for heart failure, or cardiovascular mortality. Therefore, our findings are primarily mechanistic and hypothesis-generating. They suggest a potential autonomic benefit associated with dapagliflozin pretreatment that warrants investigation in future trials designed with clinical endpoints.

### Limitations

This study has several limitations. First, this was a prospective observational study in which group allocation was determined by prior clinical use of dapagliflozin rather than randomization. Although we addressed measured confounding through multivariable adjustment, ANCOVA, and IPTW-weighted sensitivity analyses, the non-randomized design still carries an inherent risk of selection bias and residual confounding from unmeasured factors, such as subtle differences in autonomic function before treatment, physician prescribing preferences, or patient lifestyle characteristics.

Second, the sample size was relatively small, and the follow-up period was limited to three months. This may have reduced statistical power, particularly for multivariable analyses, and limited our ability to detect modest effects or to fully assess the durability of the observed HRV changes over time. In addition, the exploratory analyses of multivariable models should be interpreted cautiously. No formal *a priori* power calculation was performed, and the findings should therefore be considered preliminary.

Third, HRV is influenced by multiple physiological and behavioral factors. Despite efforts to standardize Holter monitoring conditions and daily activity instructions, day-to-day variability may still have introduced measurement noise. In addition, only a single baseline assessment was obtained, and repeat baseline testing was not performed. Future larger-scale studies with longer follow-up and more comprehensive CAN assessment methods, such as CARTs or ^123I-MIBG scintigraphy, would help further clarify the effect of SGLT2 inhibitors on cardiac autonomic function.

Fourth, the study included different GLP-1 receptor agonists, which may have varying chronotropic and autonomic effects. Because of the limited sample size, stratified analyses according to GLP-1 RA type were not feasible. Therefore, our findings should be interpreted as reflecting the overall treatment context of GLP-1 RA use in this cohort rather than definitive agent-specific effects.

Fifth, this study focused on HRV parameters as mechanistic surrogate markers of cardiac autonomic function and did not assess hard clinical outcomes such as arrhythmic events, hospitalization for heart failure, or cardiovascular mortality. Accordingly, although the findings suggest that baseline dapagliflozin use may be associated with attenuation of GLP-1 RA-related HRV decline, the clinical significance of these changes remains uncertain and requires confirmation in future studies designed with clinical endpoints.

## Data Availability

The original contributions presented in the study are included in the article/**Supplementary Material**. Further inquiries can be directed to the corresponding authors.

## Supplementary material

Supplementary Table 1 Full coefficient estimates from multivariable linear regression models for changes in HRV outcomes.
