# Supplementary material for: Dapagliflozin associates with heart rate variability decline in T2DM patients on GLP-1 receptor agonist therapy: a prospective observational study
Source: Front Endocrinol (Lausanne). 2026 May 11;17:1809146. doi: 10.3389/fendo.2026.1809146 (PMC13198993; doi:10.3389/fendo.2026.1809146)
Supplement: Supplementary Table 1 — Full coefficient estimates from multivariable linear regression models for changes in HRV outcomes. [file Table1.docx]

## Supplementary Appendix

This Supplementary Appendix provides the full multivariable regression output supporting the exploratory analyses presented in the main manuscript.

Supplementary Table S1. Full coefficient estimates from multivariable linear regression models for changes in HRV outcomes

| **Term** | **Estimate** | **Std. Error** | **Statistic** | **P value** | **95% CI lower** | **95% CI upper** |
| --- | --- | --- | --- | --- | --- | --- |
| **Outcome:** Δ **SDNN (ms)** | | | | | | |
| Intercept | 173.275 | 137.370 | 1.261 | 0.221 | -112.402 | 458.951 |
| DAPA | 30.634 | 14.023 | 2.185 | 0.040 | 1.472 | 59.795 |
| Baseline SDNN | -0.613 | 0.218 | -2.805 | 0.011 | -1.067 | -0.159 |
| Age, years | -0.347 | 0.597 | -0.582 | 0.567 | -1.589 | 0.894 |
| Sex (male=1, female=0) | -15.106 | 12.338 | -1.224 | 0.234 | -40.764 | 10.551 |
| Diabetes duration, years | -0.505 | 1.997 | -0.253 | 0.803 | -4.658 | 3.648 |
| BMI, kg/m² | -0.615 | 2.817 | -0.218 | 0.829 | -6.473 | 5.243 |
| HbA1c, % | -7.819 | 6.742 | -1.160 | 0.259 | -21.840 | 6.202 |
| HOMA-B | -0.016 | 1.331 | -0.012 | 0.990 | -2.785 | 2.752 |
| HDL-C, mmol/L | -9.926 | 28.956 | -0.343 | 0.735 | -70.144 | 50.291 |
| **Outcome:** Δ **SDANN (ms)** | | | | | | |
| Intercept | 164.166 | 127.489 | 1.288 | 0.212 | -100.961 | 429.293 |
| DAPA | 27.939 | 13.548 | 2.062 | 0.052 | -0.237 | 56.114 |
| Baseline SDANN | -0.578 | 0.270 | -2.144 | 0.044 | -1.138 | -0.017 |
| Age, years | -0.095 | 0.675 | -0.141 | 0.889 | -1.498 | 1.308 |
| Sex (male=1, female=0) | -18.210 | 14.991 | -1.215 | 0.238 | -49.385 | 12.965 |
| Diabetes duration, years | 0.796 | 1.949 | 0.408 | 0.687 | -3.258 | 4.849 |
| BMI, kg/m² | -2.389 | 2.614 | -0.914 | 0.371 | -7.826 | 3.048 |
| HbA1c, % | -4.712 | 8.010 | -0.588 | 0.563 | -21.369 | 11.945 |
| HOMA-B | -0.232 | 1.249 | -0.186 | 0.855 | -2.830 | 2.366 |
| HDL-C, mmol/L | -6.862 | 24.570 | -0.279 | 0.783 | -57.958 | 44.234 |
| **Outcome:** Δ **RMSSD (ms)** | | | | | | |
| Intercept | 30.051 | 64.288 | 0.467 | 0.645 | -103.643 | 163.745 |
| DAPA | 5.141 | 7.275 | 0.707 | 0.488 | -9.989 | 20.272 |
| Baseline RMSSD | -0.505 | 0.332 | -1.522 | 0.143 | -1.195 | 0.185 |
| Age, years | 0.134 | 0.547 | 0.244 | 0.809 | -1.003 | 1.270 |
| Sex (male=1, female=0) | -7.600 | 9.582 | -0.793 | 0.437 | -27.528 | 12.327 |
| Diabetes duration, years | 0.490 | 0.944 | 0.519 | 0.609 | -1.474 | 2.454 |
| BMI, kg/m² | 1.065 | 1.686 | 0.632 | 0.534 | -2.442 | 4.573 |
| HbA1c, % | -5.371 | 5.625 | -0.955 | 0.351 | -17.069 | 6.327 |
| HOMA-B | 0.424 | 0.628 | 0.675 | 0.507 | -0.883 | 1.731 |
| HDL-C, mmol/L | -7.536 | 17.919 | -0.421 | 0.678 | -44.802 | 29.729 |
| **Outcome:** Δ **pNN50 (%)** | | | | | | |
| Intercept | -5.050 | 13.313 | -0.379 | 0.708 | -32.735 | 22.635 |
| DAPA | 2.568 | 1.816 | 1.415 | 0.172 | -1.207 | 6.344 |
| Baseline pNN50 | -0.075 | 0.249 | -0.300 | 0.767 | -0.591 | 0.442 |
| Age, years | 0.126 | 0.114 | 1.104 | 0.282 | -0.111 | 0.363 |
| Sex (male=1, female=0) | 1.269 | 1.776 | 0.714 | 0.483 | -2.424 | 4.961 |
| Diabetes duration, years | -0.021 | 0.213 | -0.100 | 0.921 | -0.463 | 0.421 |
| BMI, kg/m² | 0.219 | 0.297 | 0.738 | 0.469 | -0.399 | 0.838 |
| HbA1c, % | -0.841 | 1.107 | -0.759 | 0.456 | -3.144 | 1.462 |
| HOMA-B | -0.082 | 0.165 | -0.495 | 0.626 | -0.425 | 0.262 |
| HDL-C, mmol/L | -1.628 | 2.711 | -0.600 | 0.555 | -7.265 | 4.010 |
| **Outcome:** Δ **lnLF** | | | | | | |
| Intercept | 0.702 | 3.879 | 0.181 | 0.858 | -7.365 | 8.769 |
| DAPA | -0.392 | 0.247 | -1.586 | 0.128 | -0.905 | 0.122 |
| Baseline lnLF | -0.253 | 0.177 | -1.425 | 0.169 | -0.622 | 0.116 |
| Age, years | -0.006 | 0.019 | -0.339 | 0.738 | -0.046 | 0.033 |
| Sex (male=1, female=0) | -0.240 | 0.363 | -0.661 | 0.516 | -0.994 | 0.514 |
| Diabetes duration, years | 0.057 | 0.027 | 2.125 | 0.046 | 0.001 | 0.112 |
| BMI, kg/m² | 0.053 | 0.070 | 0.757 | 0.458 | -0.092 | 0.198 |
| HbA1c, % | 0.007 | 0.203 | 0.033 | 0.974 | -0.417 | 0.430 |
| HOMA-B | 0.013 | 0.024 | 0.568 | 0.576 | -0.036 | 0.063 |
| HDL-C, mmol/L | -0.127 | 0.546 | -0.233 | 0.818 | -1.264 | 1.009 |
| **Outcome:** Δ **lnHF** | | | | | | |
| Intercept | 3.514 | 3.775 | 0.931 | 0.362 | -4.336 | 11.364 |
| DAPA | 0.533 | 0.332 | 1.605 | 0.123 | -0.158 | 1.223 |
| Baseline lnHF | -0.062 | 0.116 | -0.535 | 0.598 | -0.304 | 0.180 |
| Age, years | -0.016 | 0.018 | -0.880 | 0.389 | -0.055 | 0.022 |
| Sex (male=1, female=0) | -0.361 | 0.521 | -0.693 | 0.496 | -1.444 | 0.723 |
| Diabetes duration, years | 0.030 | 0.052 | 0.571 | 0.574 | -0.078 | 0.137 |
| BMI, kg/m² | -0.019 | 0.064 | -0.298 | 0.769 | -0.153 | 0.115 |
| HbA1c, % | -0.095 | 0.209 | -0.456 | 0.653 | -0.530 | 0.339 |
| HOMA-B | 0.004 | 0.029 | 0.133 | 0.896 | -0.056 | 0.064 |
| HDL-C, mmol/L | -0.916 | 0.723 | -1.267 | 0.219 | -2.420 | 0.588 |
| **Outcome:** Δ **lnLF/lnHF** | | | | | | |
| Intercept | -1.177 | 1.523 | -0.773 | 0.448 | -4.345 | 1.990 |
| DAPA | -0.184 | 0.105 | -1.752 | 0.094 | -0.403 | 0.034 |
| Baseline lnLF/lnHF ratio | 0.224 | 0.378 | 0.592 | 0.560 | -0.562 | 1.010 |
| Age, years | 0.005 | 0.008 | 0.673 | 0.508 | -0.011 | 0.021 |
| Sex (male=1, female=0) | 0.013 | 0.110 | 0.121 | 0.905 | -0.216 | 0.243 |
| Diabetes duration, years | 0.007 | 0.014 | 0.475 | 0.640 | -0.022 | 0.035 |
| BMI, kg/m² | 0.020 | 0.025 | 0.776 | 0.447 | -0.033 | 0.073 |
| HbA1c, % | -0.001 | 0.068 | -0.016 | 0.987 | -0.142 | 0.140 |
| HOMA-B | -0.002 | 0.012 | -0.167 | 0.869 | -0.026 | 0.022 |
| HDL-C, mmol/L | 0.212 | 0.229 | 0.929 | 0.363 | -0.263 | 0.688 |

Note: This table presents the full coefficient estimates from the multivariable linear regression models for each HRV change outcome. All models included baseline dapagliflozin use, the corresponding baseline HRV value, age, sex, diabetes duration, BMI, HbA1c, HOMA-B, and HDL-C. Estimates are shown as unstandardized regression coefficients with 95% confidence intervals.
